# The Reduced Plastid-Encoded Polymerase-Dependent Plastid Gene Expression Leads to the Delayed Greening of the *Arabidopsis fln2* Mutant

**DOI:** 10.1371/journal.pone.0073092

**Published:** 2013-09-03

**Authors:** Chao Huang, Qing-Bo Yu, Ruo-Hong Lv, Qian-Qian Yin, Gen-Yun Chen, Ling Xu, Zhong-Nan Yang

**Affiliations:** 1 Department of Biology, College of Life and Environmental Sciences, Shanghai Normal University, Shanghai, P.R. China; 2 Department of Biology, School of Life Sciences, East China Normal University, Shanghai, P.R. China; 3 National Key Laboratory of Plant Molecular Genetics, Institute of Plant Physiology and Ecology, Shanghai Institutes for Biological Science, Chinese Academy of Sciences, Shanghai, P.R. China; Justus-Liebig-University Giessen, Germany

## Abstract

In *Arabidopsis* leaf coloration mutants, the delayed greening phenomenon is common. Nonetheless, the mechanism remains largely elusive. Here, a delayed greening mutant *fln2–4* of FLN2 (Fructokinase-Like Protein2) was studied. FLN2 is one component of Transcriptionally Active Chromosome (TAC) complex which is thought to contain the complete plastid-encoded polymerase (PEP). *fln2–4* displayed albino phenotype on medium without sucrose. The PEP-dependent plastid gene expression and chloroplast development were inhibited in *fln2–4*. Besides interacting with thioredoxin z (TRX z), we identified that FLN2 interacted with another two members of TAC complex in yeast including its homologous protein FLN1 (Fructokinase-Like Protein1) and pTAC5. This indicates that FLN2 functions in regulation of PEP activity associated with these TAC components. *fln2–4* exhibited delayed greening on sucrose-containing medium. Comparison of the PEP-dependent gene expression among two complete albino mutants (*trx z* and *ptac14*), two yellow mutants (*ecb2–2* and *ys1*) and the *fln2–4* showed that *fln2–4* remains partial PEP activity. FLN2 and FLN1 are the target proteins of TRX z involved in affecting the PEP activity. Together with the data that FLN1 could interact with itself in yeast, FLN1 may form a homodimer to replace FLN1–FLN2 as the TRX z target in redox pathway for maintaining partial PEP activity in *fln2–4*. We proposed the partial PEP activity in the *fln2* mutant allowed plastids to develop into fully functional chloroplasts when exogenous sucrose was supplied, and finally the mutants exhibited green phenotype.

## Introduction

The first step in the life cycle of a plant is seedling establishment after seed germination. When seedlings are exposed to light, they synthesize chlorophyll; the cotyledons expand and turn green [1]. During this process, the etioplasts of cotyledons develop into photosynthetically functional chloroplasts, which allow seedlings to become photoautotrophy and no longer depend on energy stored in the seed [2], [3]. Disruption of chloroplast function often results in severe phenotype, such as embryo lethality, albinism, or pale-green plants [4]. Chloroplast development is regulated by both nuclear and plastid genomes, and recent investigations revealed that many factors are involved in the process [5], [6].

The transcription of plastid genes in higher plants is dependent on two RNA polymerases, the nuclear-encoded plastid RNA polymerase (NEP) and plastid-encoded plastid RNA polymerase (PEP) [7]. NEP preferentially transcribes housekeeping genes, while PEP is responsible for transcribing genes involved in photosynthesis [8], [9]. PEP and its associated proteins/DNA can be purified by different biochemical purification procedures [10], [11]. This protein/DNA-complex, termed “transcriptionally active chromosomes” (TAC), can be separated from the plastid components by gel filtration [12]–[14]. In *Arabidopsis* and mustard (*Sinapis alba*), a total of 35 components have been identified in the TAC complex including the core subunits of PEP encoded by plastome-located *rpo* genes and nuclear encoded subunits [15].

In recent years, a group of *Arabidopsis* leaf coloration mutants defective in the TAC components have been isolated. The Fe-superoxide dismutase (FSD) double mutant *fsd2–1 fsd3–1* exhibited low tolerance to oxidative stress and accumulated decreased amounts of mRNA for PEP-dependent plastid genes [16]. Studies on the knockout mutants of thioredoxin z (TRX z) and fructokinase-like protein (FLN) showed that TRX z interacts with FLN1 and FLN2 to regulate the PEP-dependent transcription and chloroplast development in redox signaling pathway [17]–[19]. Mutations in the *ptac3*, *ptac7* and *ptac14* genes also resulted in the expression defects of PEP-dependent genes [20]–[22]. Based on the decreased expression of PEP-dependent genes, the PEP activity in these leaf coloration mutants was severely impaired which led to complete albino phenotype.

However, there exists another group of delayed greening mutants including *ptac2* [15] and some non-*ptac* mutants, such as *wco* [23], *dg1* [24] and *pisp1* [25]. These mutants initially showed an albino phenotype on medium without sucrose, but they could turn green when exogenous sucrose was provided. In addition, a group of yellow mutants were reported such as *ys1* [26] and *ecb2–2* [27]. These mutants shared a similar phenotype that their cotyledons and true leaves were initially very yellow, but then turned green gradually without supplementation with exogenous carbon. *YS1* encodes a chloroplast-localized pentatricopeptide repeat (PPR) protein, which is required for the editing of *rpoB* transcripts encoding the beta subunit of PEP in *Arabidopsis* [26]. *AtECB2* also encodes a PPR protein with a C-terminal DYW domain. It regulates the editing of the plastid genes *accD* and *ndhF* [27]. The PEP activity has not been analyzed in *pisp1* [25]. In all other delayed greening mutants and yellow mutants, PEP activity was defective [15], [23], [24], [26], [27]. Nonetheless, the mechanism for the greening of these PEP-related leaf coloration mutants is largely unknown.

During the seedling greening process, one of the most important events is the formation of chlorophyll, which allows plants to absorb energy from light [28]. The chlorophyll biosynthesis is strictly regulated by environmental and endogenous cues such as light signals [29], hormone signals [30], and plastid retrograde signals [31]. Previous studies revealed that several signaling-responsive transcription factors are required for chlorophyll biosynthesis. The transposase-derived transcription factors FHY3/FAR1 are responsive to light signals and directly activate the expression of the key gene *HEMB1* in chlorophyll biosynthetic pathway [32]. The ethylene-stabilized transcription factors EIN3/EIL1 were found to promote chlorophyll synthesis in the ethylene-induced signaling pathway [33]. In addition, recent studies have demonstrated that the regulation by light, auxin/cytokinin and plastid-derived retrograde signals is dependent on the golden2-like transcription factors (GLKs), which is required for the expression of several chlorophyll biosynthesis genes [34], [35]. Apart from the known chlorophyll biosynthesis, photosynthesis gene expression is crucial for the greening process by affecting the assembly of the photosynthetic apparatus [35]. PEP is the major machinery in regulating photosynthesis-related plastid gene expression. Thus, it is necessary for us to elucidate the relationship between PEP and seedling greening.

Here, we report the characterization of a delayed greening mutant *fln2–4* in *Arabidopsis*, which displays albino phenotype but can develop greenish true leaves on sucrose-containing medium. The PEP-dependent plastid gene expression and chloroplast development were inhibited in *fln2–4*. Comparison of the PEP-dependent gene expression among five leaf coloration mutants (*trx z*, *ptac14*, *fln2–4*, *ecb2–2* and *ys1*) indicates that the PEP activity is critical for the leaf color phenotypes. The different degrees of PEP activity often give rise to the different leaf colors. Based on our yeast two-hybrid assay, FLN1 may form a homodimer instead of the FLN1–FLN2 heterodimer to function in regulation of PEP activity, which supports that the PEP activity in *fln2–4* is higher than that in the complete albino mutants (*trx z* and *ptac14*). The relatively high PEP activity in *fln2–4* allows the slow accumulation of the PEP-dependent gene transcripts for chloroplast development when supplemented with sucrose. With the formation of functional chloroplasts, the *fln2–4* mutant shows green phenotype. All of these investigations should prove to be helpful to understand the mechanism of the greening phenomenon in many PEP-related *Arabidopsis* leaf coloration mutants.

## Results

### Identification and Characterization of the *fln2* Mutant

**Figure 1:**
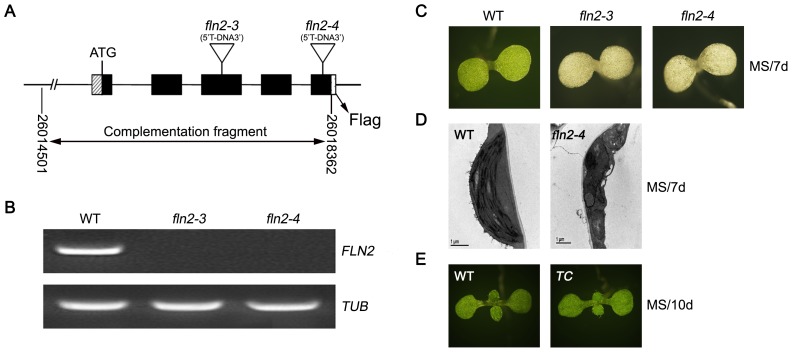
T-DNA insertion sites of *fln2* and their phenotypes. (A) The sketch map of the *FLN2* gene, the T-DNA insertion sites of *fln2* mutants. Boxes, exons; lines, introns. (B) The expression of *FLN2* in WT (Col-0), *fln2–3* and *fln2–4*. (C) Photographs of the *fln2–3* and *fln2–4* seedlings grown on MS medium for 7 days. (D) Ultrastructural analysis of chloroplasts from 7-day-old WT and *fln2–4* seedlings grown on MS medium. Scale bars: 1 µm. (E) Phenotypes of WT and the *fln2–4* complemented seedlings grown in soil.

To analyze the functional roles of *FLN2* gene during plant growth and development, we obtained two T-DNA insertion lines, SALK_005734 and CS811853, from the Arabidopsis Biological Resource Center (ABRC, http://abrc.osu.edu/). In these two lines, the T-DNA was inserted in the 3^rd^ exon and the 5^th^ exon of the *FLN2* gene, respectively (Figure 1A). Reverse transcription-polymerase chain reaction (RT-PCR) analysis showed that the *FLN2* transcript was absent in both mutants (Figure 1B). Due to the SALK_005734 line named as *fln2–3* [19], the CS811853 line in this work was termed as *fln2–4*. Both *fln2–3* and *fln2–4* displayed albino cotyledons and were seedling lethal on MS medium without sucrose (Figure 1C). The phenotypes of the two allelic mutants were similar; thereby the *fln2–4* mutant was chosen for further analysis. Transmission electron microscopy (TEM) observations revealed that the chloroplasts in the 7-day-old *fln2–4* mutants had a visible change in ultrastructural organization with irregular morphology and lacked internal membrane structures (Figure 1D). To confirm that the knockout of *FLN2* was responsible for the defects in the *fln2–4* phenotype, a construct containing the genomic sequence of the *FLN2* gene, as well as 1517-base pair (bp) upstream and the FLAG sequence was introduced into the heterozygous plant (*FLN2/fln2–4*). A total of 53 transgenic plants were obtained. Six of them were identified to be homozygous for the T-DNA insertion, and exhibited normal morphology as the wild type (WT) (Figure 1E). These results demonstrate that the *FLN2* gene is responsible for the defective phenotype in *fln2–4* mutant, and *FLN2* is important for chloroplast development and seedling growth.

### Loss of *FLN2* Down-Regulates the Expression of PEP-Dependent Plastid Genes

**Figure 2:**
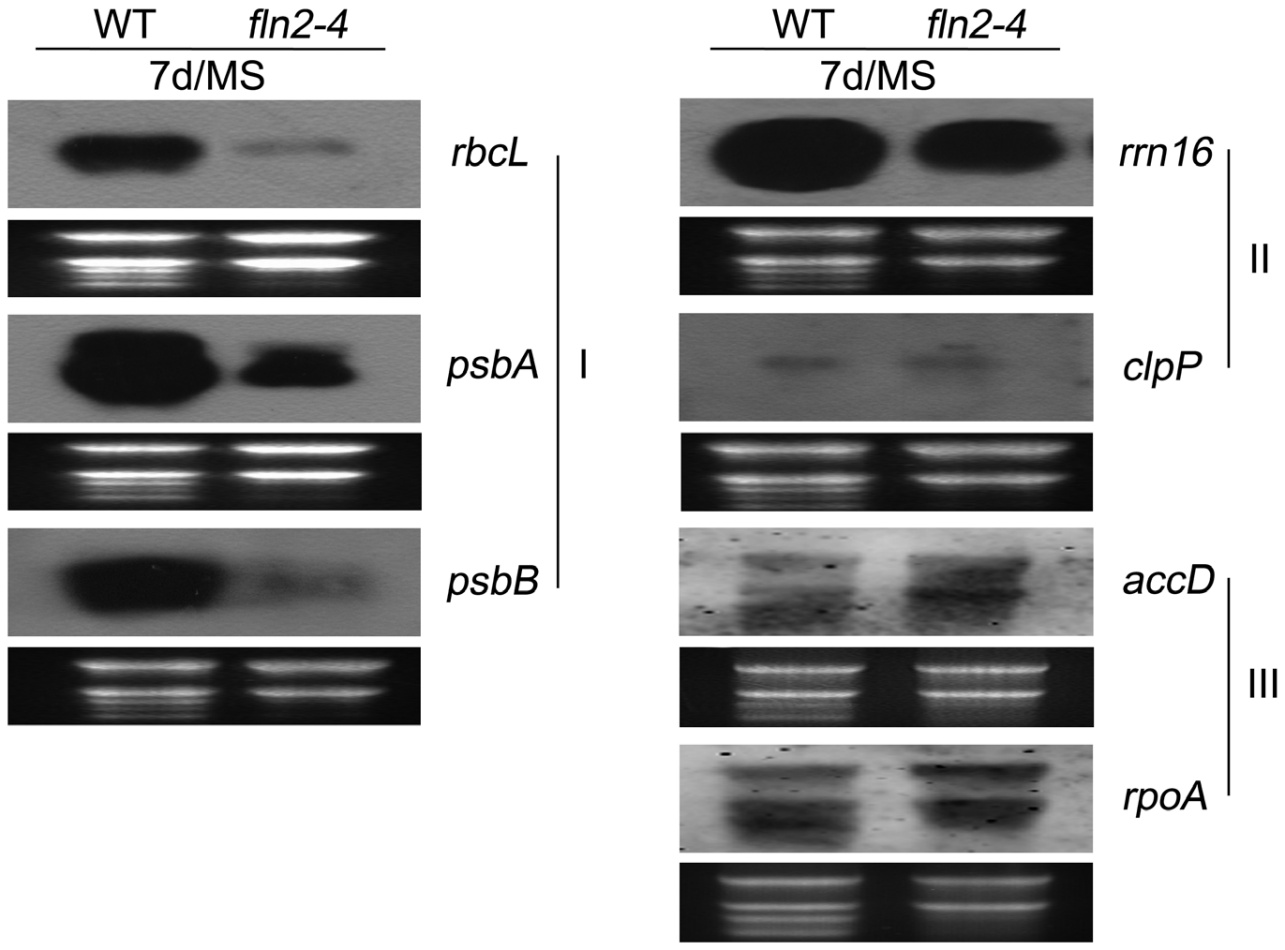
Expression analysis of the plastid encoded genes in *fln2–4* seedlings. Northern blot was performed to detect the plastid gene transcript levels in the 7-day-old *fln2–4* seedlings and WT grown on MS medium without sucrose. Three classes of genes were examined, *psbA, psbB*, and *rbcL* were selected as PEP-dependent genes, *clpP* and *rrn16* were selected as PEP- and NEP-dependent genes, *accD* and *rpoA* were selected as NEP-dependent genes.

FLN2 is one component of TAC, and many TAC members were reported to affect the plastid gene expression [15]–[17], [20]–[22]. To investigate the effect of the knockout of the *FLN2* gene on the plastid gene expression, we used Northern hybridization to examine the transcriptional levels of plastid genes in 7-day-old *fln2–4* mutants grown on MS medium without sucrose. The plastid genes are categorized into three classes based on whether they are transcribed by PEP and/or NEP [36]. The class I and class III genes are exclusively transcribed by PEP and NEP, respectively. While the class II genes are both PEP- and NEP-dependent. Our results showed that the expression of the class I genes (*psbA, psbB* and *rbcL*) were strongly reduced in the *fln2–4* mutant compared with that of the WT. By contrast, the transcript levels of the class-II genes (*rrn16* and *clpP*) and the class-III genes (*accD* and *rpoA*) did not have any significant variation between *fln2–4* mutant and WT (Figure 2). These results confirm that loss of *FLN2* mainly affects the expression of PEP-dependent plastid genes.

### FLN2 Interacts with FLN1 and pTAC5 in Yeast

**Figure 3:**
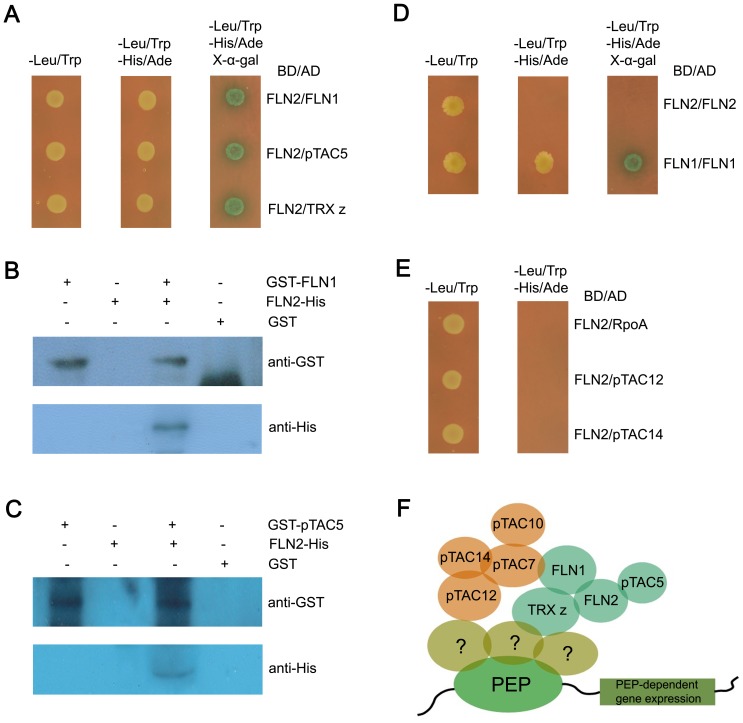
The relationships between FLN2 and other components of TAC. (A) Interactions of FLN2 with FLN1, pTAC5 and TRX z proteins in yeast. (B) GST pull-down assay revealed the existence of a physical interaction between FLN2 and its homologous protein FLN1. (C) In vitro GST pull-down assay for interaction between FLN2 and pTAC5. (D) FLN1 can interact with itself in yeast, while FLN2 can not. (E) Non-interaction existed between FLN2 and three essential subunits of TAC complex including RpoA, pTAC12 and pTAC14. (F) The primary working model for TRX z, FLN1, FLN2, pTAC5, pTAC7, pTAC10, pTAC12 and pTAC14.

FLN2 belongs to the components of TAC complex in *Arabidopsis* [15]. To establish the relationship between FLN2 and the other components of TAC, we performed a yeast two-hybrid screen. FLN2 was fused to the GAL4 DNA-binding domain (BD) as a bait to screen a pool composed of the thirty-five reported TAC components fused to the GAL4 activation domain (AD). After yeast transformation, we randomly selected clones with blue appearance in selective dropout (SD) medium lacking trptophan (Trp), leucine (Leu), histidine (His) and adenine hemisulfate salt (Ade) with X-α-gal for PCR amplification. Sequencing the PCR products identified three genes including *FLN1*, *pTAC5* and *TRX z*. Yeast two-hybrid experiments verified the interactions between FLN2 and the three proteins (Figure 3A). We subsequently performed pull-down assay to further confirm the interactions between FLN2 and FLN1 or pTAC5. Results showed that both the recombinant proteins GST-FLN1 and GST-pTAC5 were able to pull down FLN2-His (Figure 3B and C). FLN2 and FLN1 belong to the pfkB family, and share high peptide similarity [37]. To examine whether the FLN1 and FLN2 proteins form homodimers, we analyzed the interactions between them by yeast two-hybrid assay. Results showed that FLN1 can interact with itself, but FLN2 can not (Figure 3D). RpoA, pTAC12 and pTAC14 are the essential subunits in the PEP complex [18]. Yeast two-hybrid assay showed that FLN2 did not interact with these essential subunits in yeast (Figure 3E). Based on previous investigations [21], [22] and data in this study, a working model for partial TAC components is proposed (Figure 3F). In this model, pTAC7 interacts with FLN1, pTAC10, pTAC12 and pTAC14 while FLN2 interacts with FLN1, TRX z and pTAC5.

### The *fln2–4* Mutant Exhibited a Delayed Greening Phenotype when Grown on Sucrose-Containing Medium and can Grow Autotrophically in Soil

**Figure 4:**
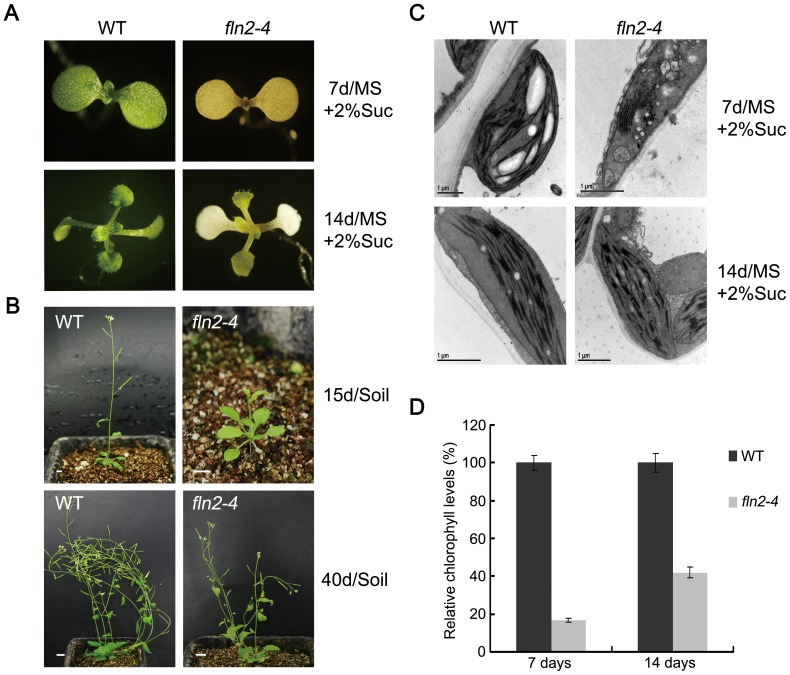
Alterations to seedling development in *fln2–4* plants. (A) The phenotypes of WT and *fln2–4* plant grown on 2% sucrose-containing MS medium for 7 days and 14 days. (B) The phenotypes of WT and *fln2–4* plants grown in soil after growing on sucrose-containing medium for 14 days. Bars represent 1 cm. (C) Chloroplast ultrastructure in 7-day-old WT, 7-day-old *fln2–4* plants, 14-day-old WT and 14-day-old *fln2–4* plants. All of these plants grew on sucrose-containing medium. Scale bars: 1 µm. (D) The relative chlorophyll levels in WT and *fln2–4* seedling during growth on sucrose-containing MS medium. The values presented are averages of three replicates ± SD.

**Figure 5:**
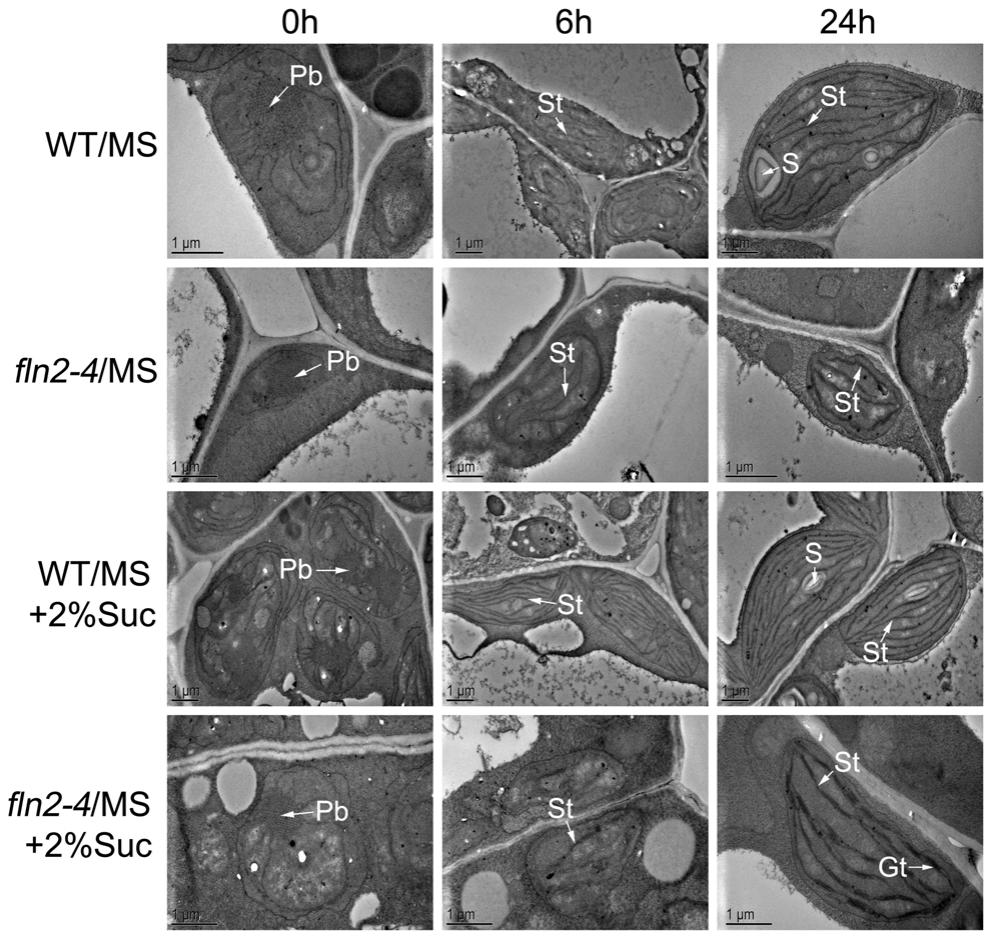
Ultrastructure development of WT and *fln2–4* cotyledon plastids during de-etiolation. Seedlings were grown in darkness for 5 days, and subsequently illuminated for 0 h (left panels), 6 h (middle panels) and 24 h (right panels). Abbreviations used in the panels are listed: Pb, prolamellar body; S, starch granule; St, stromal thylakoids; Gt, grana thylakoids. Scale bars: 1 µm.

The delayed greening *fln2–4* mutant exhibits sugar-dependent for the survival of seedlings and growth of green true leaves. When supplemented with sucrose, the 7-day-old *fln2–4* seedlings displayed yellow-tinted cotyledons, and then developed greenish true leaves in two weeks after germination (Figure 4A). After transplanting them into soil, all the mutant seedlings with greenish true leaves could flower and produce fertile seeds without sucrose supplementation (Figure 4B). To unveil the ultrastructural basis of *fln2–4* seedlings during the delayed greening process, we examined dynamic changes in chloroplasts using TEM. In the leaves of 7-day-old WT grown with sucrose, chloroplasts were crescent-shaped and contained well-formed internal membrane structures including stroma thylakoids and stacked grana thylakoids (Figure 4C). In contrast, at the same stage, the chloroplasts in the 7-day-old *fln2–4* mutants were still highly vacuolated, but the lamellar membrane structures appeared (Figure 4C). In the 14-day-old *fln2–4* mutants grown under the same conditions, chloroplasts with well-organized thylakoid membrane could be observed (Figure 4C). This suggests the *fln2–4* chloroplast can be gradually formed although its development is slower than that in WT. In addition, we measured the levels of chlorophyll a and b in both the mutant and WT seedlings during the greening process. The total chlorophyll content of *fln2–4* mutant (93.58±2.45 mg g^−1^ fresh weight for the *fln2–4* mutant type) was about 16.9% of WT (554.94±15.51 mg g^−1^ fresh weight for the wild type) grown with sucrose for 7 days, and the chlorophyll a/b ratio of *fln2–4* was about half of WT (Figure 4D; Table 1). In 14-day-old mutant seedlings grown under the same conditions, the chlorophyll content (292.16±24.91 mg g^−1^ fresh weight for the *fln2–4* mutant type) was about 42% compared to that of the WT (695.19±24.14 mg g^−1^ fresh weight for the wild type), and the chlorophyll a/b ratio was close to that of WT (Figure 4D; Table 1). These observations indicated that the chlorophyll biosynthesis in *fln2–4* was partially recovered. Furthermore, we investigated the plastid ultrastructure development of the WT and *fln2–4* grown on sucrose-containing medium during de-etiolation. In the WT and *fln2–4* seedlings grown in darkness for 5 days, the etioplasts contained a large prolamellar body (Figure 5, left panels). When etiolated seedlings were exposed to light for 6 hours, the prolamellar in WT developed into stromal lamellae (Figure 5, middle panels). In contrast, plastids in *fln2–4* contained less stromal lamellae (Figure 5, middle panels). After de-etiolation for 24 hours, plastids in WT contained well-developed thylakoid membrane system and starch granules, while loose thylakoid lamellae and grana thylakoids were detected in *fln2–4* (Figure 5, right panels). When grown on MS medium without sucrose in darkness for 5 days, the etioplasts of *fln2–4* were similar with that of the WT. After de-etiolation for 24 hours, only a few thylakoid lamellae could be observed in *fln2–4* (Figure 5). These results revealed that plastid development in *fln2–4* grown on sucrose-containing medium proceeds slowly, but it can gradually form well-structured chloroplast during the de-etiolation process.

**Table 1:** Chlorophyll accumulation in the WT and *fln2–4* mutants.

Sample	mg Chl(a+b)/g FW*	Relative amount	Chl a/b ratio
WT 7 d/MS+2%Suc	554.94±15.51	100%	2.03±0.02
*fln2–4* 7 d/MS+2%Suc	93.58±2.45	16.9%	0.92±0.13
WT 14 d/MS+2%Suc	695.19±24.14	100%	1.94±0.07
*fln2–4* 14 d/MS+2%Suc	292.16±24.91	42.03%	1.53±0.18

* Averages ± standard deviations of chlorophyll (Chl) concentrations for 3 independent measurements. FW: fresh weight.

### PEP-Dependent Plastid Gene Expression in *fln2–4* Is Higher than that in the Complete Albino Mutants, but Lower than that in the Yellow Mutants

**Figure 6:**
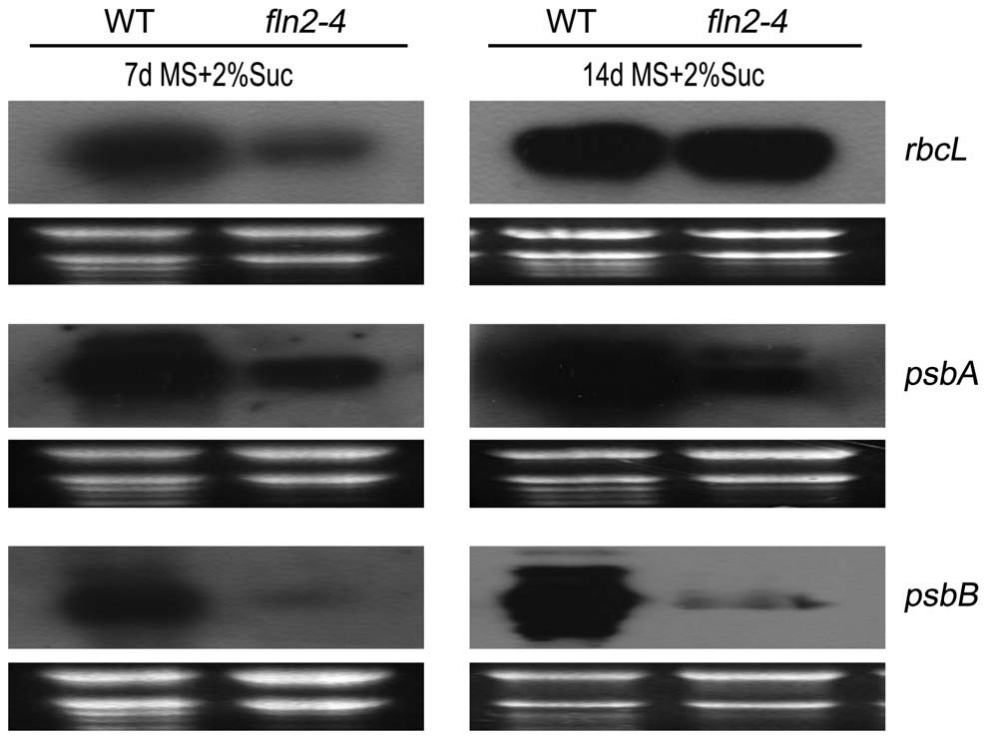
Changes in the transcript levels of PEP-dependent genes during the greening process of *fln2–4* mutant. The expression levels of *rbcL*, *psbA* and *psbB* genes in 7-day-old and 14-day-old *fln2–4* mutants were determined by Northern blot as compared with WT, respectively. The experimental WT and *fln2–4* seedlings were grown on sucrose-containing MS medium.

**Figure 7:**
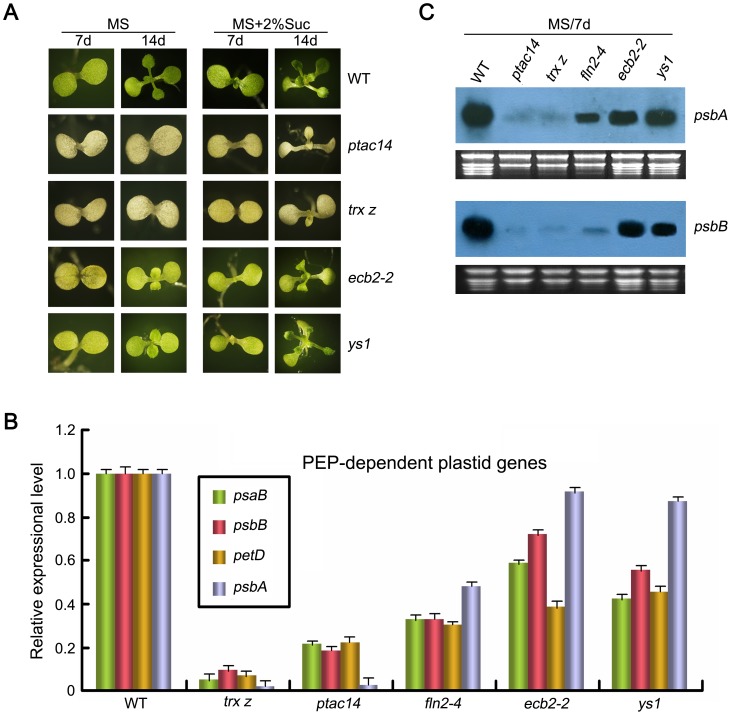
Comparison of PEP-dependent plastid gene expression between *fln2–4* and four leaf coloration mutants. (A) The phenotypes of WT and four leaf coloration mutants grown with or without sucrose. (B) qRT-PCR analysis the transcript levels of four PEP-dependent plastid genes in *fln2–4* seedlings and other four leaf coloration mutants grown on MS medium without sucrose for 7 days. These PEP-dependent genes refer to *psbA*, *psbB*, *psaB*, *petD*. Expression levels are presented as the percentage relative to WT. Data are means ± SD (n = 3). (C) The accumulation of *psbA* and *psbB* transcripts detected by Northern blot.

The most distinct characteristic of *fln2–4* mutant is its delayed greening phenotype when grown on sucrose-containing medium. We assayed the expression of PEP-dependent plastid genes in *fln2–4* during its greening process. Results showed that the transcript levels of the three selected genes (*rbcL*, *psbA*, *psbB*) in 7-day-old *fln2–4* mutant were still significantly lower than that in WT. However, after 14 days of growth on sucrose-containing medium, the *rbcL* mRNA accumulated to similar levels in WT, while the *psbB* transcription was slightly enhanced (Figure 6). These results indicated that the transcripts of several PEP-dependent genes were able to gradually accumulate during the greening of *fln2–4*. To disclose whether these differences in plastid gene expression were tightly linked with leaf phenotype, we chose four leaf coloration mutants for further research, which including two complete albino mutants *trx z* [17] and *ptac14* [22], and two yellow mutants *ecb2–2* [27] and *ys1* [26]. When germinated on MS medium, *trx z* and *ptac14* exhibited albino cotyledons and died before developing true leaves. While grown on MS medium containing 2% sucrose, they could produce pale yellow cotyledons and true leaves (Figure 7A). Nevertheless, these mutants subsequently died. In contrast, the *ecb2–2* and *ys1* seedlings initially exhibited yellow cotyledons and were able to turn green without exogenous sucrose (Figure 7A). However, the mature *ecb2–2* and *ys1* plants were slightly weaker than WT (data not shown). We compared the PEP activity in these four leaf coloration mutants (*trx z*, *ptac14*, *ecb2–2* and *ys1*) and the *fln2–4* through detecting the transcript abundance of four PEP-dependent chloroplast genes (*psaB*, *psbA*, *psbB* and *petD*). Quantitative Real-Time PCR (qRT-PCR) assay showed that the expression of these genes in the two complete albino mutants (*trx z* and *ptac14*) were lower than that in the *fln2–4*, *ecb2–2* and *ys1*, respectively. In addition, the expression of these genes in the delayed greening *fln2–4* mutant was lower than that in the yellow mutant *ecb2–2* and *ys1* (Figure 7B). Northern blot analysis using probes for *psbA* and *psbB* in these mutants exhibited the similar result (Figure 7C). All of these results suggest that the PEP activity in *fln2–4* is higher than that in the complete albino mutants but lower than that in the yellow mutants.

## Discussion

### FLN2 is One Component of TAC to Regulate PEP-Dependent Plastid Gene Expression

The knockouts of many TAC members in *Arabidopsis* have been reported to affect plastid gene expression and chloroplast development including *ptac6*, *ptac12* [15], *fsd2*, *fs*d3 [16], *trx z* [17], *fln1* [17], [18], *ptac14* [18], [22], *ptac3* [20] and *ptac7* [21]. FLN2 is one component of TAC and is involved in the redox pathway mediated by TRX z [15], [17]. The knockout lines *fln2–1* and *fln2–2*, exhibited pale green cotyledons and slow greening on growth medium and soil [19]. Here, we isolated another two knockout lines *fln2–3* and *fln2–4*. These two lines displayed albino phenotype when grown on MS medium (Figure 1C), but they could turn green when grown on sucrose-containing medium (Figure 4A and data not shown), which was slightly different from the reported phenotypes of *fln2–1* and *fln2–2* [19]. This difference was probably due to the different growth conditions. Nevertheless, the PEP-dependent plastid gene expression was down-regulated in all the four lines (Figure 2) [19], as well as in the *Arabidopsis* RNAi plants [17]. Therefore, FLN2 is similar with other functional TAC components in regulating the PEP-dependent plastid gene expression and chloroplast development.

TRX z interacts with FLN1 and FLN2 [17], and recently pTAC7 has been reported to interact with FLN1, pTAC10, pTAC12 and pTAC14 [21]. Besides interacting with TRX z, we found that FLN2 also interacts with FLN1 and pTAC5 in yeast (Figure 3A). These interactions revealed the preliminary relationship of these TAC components in the complex (Figure 3F). The TAC components appear to perform basic functions or play roles as regulators for the transcription regulation and environmental adaptation in chloroplast. Steiner et al. identified 10 essential non-*rpo* subunits of the PEP complex named PEP-associated proteins (PAPs) [18]. These PAPs might be the core subunits of the TAC complex and play the basic function in regulation of plastid gene expression. FLN1 and TRX z belong to PAPs, while the FLN2 and pTAC5 were not included. The *fln1* [18], [19] and *trx z* (Figure 7A) displayed seedling lethality, which were more severe than that of *fln2*. In addition, silencing of FLN2 results in weaker effect on PEP-dependent plastid gene expression than does silencing of FLN1 [17]. Furthermore, the *ptac5* mutant showed similar phenotype with WT under normal condition, but was sensitive to heat stress (Figure S1). Overall, FLN2 and pTAC5 may represent peripheral component rather than core subunit to function as a regulator for environmental adaptation in the TAC complex.

### PEP Activity is Critical for the Early Development of Chloroplast and Leaf Greening

Leaf coloration mutants are frequently observed in plant. In *Arabidopsis*, seedling leaf coloration mutants can be classified into several groups. For the yellow mutants such as *ys1* [26] and *ecb2–2* [27], they initially exhibited yellow cotyledons and true leaves, and subsequently turned green and grew photo-autotrophically under normal condition. For the complete albino mutants such as *trx z* [17] and *ptac14* [22], they displayed albino cotyledons and could not turn green even on sucrose-containing medium (Figure 7A). These lines are seedling lethal and can not complete their life cycles. Apart from the complete albino mutants and the yellow mutants, there exists another group of delayed greening mutants such as *ptac2* [15], *wco* [23], *dg1* [24] and *pisp1* [25]. These mutants showed albino phenotype initially, however, they could turn green on the sucrose-containing medium. The *fln2–4* mutant is similar with the delayed greening mutants (Figure 4A). These different groups of leaf coloration mutants often showed different degrees of defects in chloroplast development. During seed germination, the photosynthetically functional chloroplasts are quickly formed and seedlings turn green before the energy in the seed is exhausted. The rapid formation of mature chloroplast during seed germination is very important for seedling greening. Thus, it seems that the different degrees of chloroplast developmental status give rise to the different leaf colors of mutant seedlings.

The PEP-dependent plastid gene expression analysis in this work indicates the PEP activity in the delayed greening mutant is higher than that in the complete albino mutants, but lower than that in the yellow mutants (Figure 7B and C). The PEP activity in these leaf coloration mutants is in agreement with their phenotypes. The more seriously reduced PEP activity results in the more severe phenotype. Recently, Zhelyazkova et al. have reported that PEP is the dominating RNA polymerase in chloroplast maturation [38]. Based on their work together with the data in this paper, we hypothesize that a threshold of PEP activity is critical for the chloroplast development and leaf greening. In complete albino mutants, the PEP activity is below-threshold and the chloroplast development in these mutants may be completely blocked. In the *fln2–4* mutant, relatively high PEP activity allows the slow chloroplast development. However, the energy in seed is not enough to support the formation of mature chloroplast and therefore seedlings show albino phenotype. The exogenous sucrose in the medium supports the mutant plants to overcome energy deficiency and gradually accumulate enough photosynthesis gene transcripts for functional chloroplast formation. The development of fully functional chloroplast allows the mutant plants to show green phenotype.

### A Homodimer Formed by FLN1 may Partially Compensate the Loss of FLN2–FLN1 Heterodimer to Maintain some PEP Activity

The cellular redox environment has a fundamental role in regulating most plastid processes, including secondary metabolism, gene transcription, and protein synthesis as well as import [39], [40]. In *Arabidopsis*, Arsova et al. identified that a novel thioredoxin TRX z which may be involved in the redox pathway to regulate PEP-dependent plastid gene expression and chloroplast development through interacting with FLN1 and FLN2 [17]. Here, our data showed that there exist an interaction between FLN1 and FLN2, which indicated that the FLNs may form a heterodimer as the TRX z target in the TAC complex. The homologs of FLN1 and TRX z are present in moss, pteridophyta and angiosperm, while FLN2 homologs are only present in angiosperm, based on the available genomic data. This suggests that FLN1 might be an ancient protein, while FLN2 is a novel protein evolved from FLN1 after the emergence of the pteridophyta during the course of evolution. In this work, FLN1 was found to interact with itself in yeast (Figure 3D). This suggests that FLN1 might form a homodimer in the TAC complex to regulate the PEP activity and chloroplast development in moss. With the presence of FLN2, the FLN1 may interact with FLN2 rather than itself. However, as the essential protein in the redox pathway, FLN1 remains the ability to interact with itself. In *Arabidopsis*, both the TRX z and FLN1–FLN2 heterodimer are the essential components of redox pathway in regulation of PEP activity. The PEP activity in the knockout of TRX z was severely impaired and therefore the *trx z* mutant showed complete albino phenotype. In the *fln1* mutant, the redox pathway may be blocked due to the absence of the FLN1–FLN2 heterodimer, which leads to albinism phenotype. While in the *fln2–4* mutant, FLN1 may form a homodimer to replace the FLN1–FLN2 heterodimer, and thus the mutant remains partial signaling of the redox pathway in regulation of PEP activity. This supports the slow accumulation of the PEP-dependent plastid gene transcripts for chloroplast development and the delayed greening of this mutant.

## Materials and Methods

### Plant Materials and Growth Conditions

WT and mutant *Arabidopsis thaliana* (*Columbia*) seeds were used in this study. The T-DNA insertion lines SALK_005734 (*fln2–3*), CS811853 (*fln2–4*), SALK_0028162 (*trx z*), SALK_005814 (*ptac14*), SALK_049133 (*ptac5*) were obtained from the ABRC. The *ecb2–2* mutant was screened using an ethylmethane sulfonate mutagenesis strategy as described in our recent paper [27]. Plants were grown on Murashige and Skoog (MS) agar plates supplemented with or without 2% (w/v) sucrose. All plate-grown seedlings were vernalized under 4°C condition for 2 days and then were grown in a chamber at 22°C with a 16-h-light (120 mmol photonsm^–2^ s^–1^)/8-h-dark cycle. The soil-grown plants were grown with a photon flux density of 120 mmol m^–2^ s^–1^ at 22°C.

### Identification and Complementation of the *fln2* Mutant

The primers used for PCR verification of the T-DNA insert in the mutant were AtLB1 (5′-TGGTTCACGTAGTGGGCCATCG-3′) and the plant specific primers (*fln2–3*-LP, 5′-GAGATTTTCATGCCAAAGCTG-3′; *fln2–3-*RP, 5′-CAGCTTCTTCTGATGTGGAGG-3′; *fln2–4-*LP, 5′-TTGGAACATTGAGTTTTTGGC-3′; *fln2–4-*RP, 5′-TCATCGTCACTGCAGTTTCAC-3′). The abovementioned primers also were used to distinguish the etiolated WT and *fln2–4* seedlings during de-etiolation process. A 3861-bp DNA fragment including the genomic sequence of the gene *FLN2* (At1g69200) and 1517-bp upstream sequence, was amplified using KOD polymerase (Takara, Japan) with the two specific primers (*FLN2*-F: 5′-GGATCCAATTATTCATTCTGTTTCCAACATTGT-3′; *FLN2*-R: 5′-GTCGACTAAACTACCATCTTCAAACATTGAGCC-3′). The fragment was subcloned into pCAMBIA1300-3×FLAG vector (modified from pCAMBIA1300) and then introduced into heterozygous *FLN2/fln2–4* plants by *Agrobacterium tumefaciens*-mediated transformation. The genomic background of these independent hygromycin-resistant transgenic plants was verified by PCR analysis with a primer of AtLB1 and the plant-specific primers (*FLN2*-F: 5′- CAGCTTTGGCATGAAAATCTC-3′ and *FLN2-*R, 5′-CCAGAGGATCTAGCCCTTGAG-3′).

### Chlorophyll Determination and Chloroplast Ultrastructure

Total chlorophyll content was measured according to the record as described earlier [41]. *Arabidopsis* leaf segments were from the cotyledons of 7-day-old *fln2–4* plants grown with or without sucrose and the true leaves of 14-day-old mutants grown on sucrose-containing medium. Transmission electron micrographs were obtained exactly as described earlier [42]. The specimens were examined Hitachi H7650 transmission electron microscopy (http://www.hitachi.com).

### RNA Isolation, cDNA Synthesis, RT-PCR Analysis and Quantitative Real-Time RT-PCR

Procedures for the purification of total RNAs for cDNA synthesis, RT-PCR, and qRT-PCR were performed as previously described [41]. The specific primers used to quantify the expression of *FLN2* were as follows: sense primer, 5′-ATGGCTGCTGGTAGGAGAAAG-3′; antisense primer, 5′- TCATAAACTACCATCTTCAAA-3′. The β-tublin was used as the internal standard for qRT-PCR analysis.

### Northern Hybridization

Approximately 10µg total RNAs of each sample were separated on a 1.5% formaldehyde-agarose gel, transferred onto a nylon membrane (Pall, Mexica) and hybridized with the specific probes. The probes were synthesized with a PCR amplification-DIG-labeling kit. Sequence data for the PCR primers performed in this study can be found in previous report [22]. Chemiluminescent detection was carried out according to the Roche manual (Roche, http://www.roche.com).

### Yeast Two-Hybrid Assays

Yeast two-hybrid techniques were performed as described [22]. The following gene-specific primers were used: the *FLN1* gene cloned into pGADT7 and pGBKT7 vector were amplified using primers 5′-CCCGGGCATGGCTTCAATTAATGGCAGC-3′ and 5′-CTCGAGCTACCACATTGATGGAACATA-3′, 5′- GAATCCATGGCTTCAATTAATGGCAGC-3′ and 5′- GTCGACCTACCACATTGATGGAACATA-3′, respectively. The *FLN2* gene cloned into pGADT7 and pGBKT7 vector were amplified using primers 5′-GGATCCGC ATGGCTGCTGGTAGGAGAAAG-3′ and 5′-GAGCTC TCATAAACTACCATCTTCAAA-3′, 5′-CCATGG TGATGGCTGCTGGTAGGAGAAAG-3′ and 5′- GGATCCTCATAAACTACCATCTTCAAA-3′, respectively. The *pTAC5* and *rpoA* gene cloned into pGADT7 vector were amplified using primers 5′-CATATGATGTGCTTCTCCACTCAAAATC-3′ and 5′-GGATCCTTATAAGTTTTTTTTGCCGTC-3′, 5′-GAATCCATGAATAACTTTGAAGACAGA-3′ and 5′-GGATCCCTATTTTTTTTCTAGAATGTC-3′, respectively. The primers for *pTAC12* and *pTAC14* can be found in previous report [22].

### Protein Expression and GST Pull-Down Experiment

The full-length *FLN1* and *pTAC5* lacking the N-terminal transit peptide sequence were cloned into pGEX-4T-1(GE Healthcare, London, UK), and the full-length *FLN2* without transit sequence was cloned into pET51b (Novagen, Merck, Darmstadt, Germany) vector. The primers used to amplify for *FLN1*, *FLN2* and *pTAC5* were as follows: 5′-GTCGACTCATGGCTTCAATTAATG-3′ and 5′- GCGGCCGCCACATTGATGGAACATA-3′, 5′-GGATCCG ATGGCTGCTGGTAGGAG-3′ and 5′-GAGCTCTAAACTACCATCTTCAA-3′, 5′- GGATCCATGTGCTTCTCCACTCAAAATC-3′ and 5′-GTCGAC TAAGTTTTTTTTGCCGTCGCA-3′, respectively. These proteins were overexpressed in *Escherichia coli* BL21 (DE3) pLysS (Promega, Madison, Wisconsin, USA) strain for 6 hours at 28°C, and then the His-fused and the glutatione S-transferase (GST)-fused proteins were incubated with 40 µl glutathione sepharose 4B bead (GE Healthcare, London, UK) for 2 hours at 4°C. Pulled-down proteins were extensively washed with buffer containing 20 mM Tris-HCl, 0.1mM ethylenediaminetetraacetic acid (EDTA), 100 mM NaCl, and 0.2% Triton X-100, pH 7.4 before the samples were resolved on 12% sodium dodecyl sulfate-polyacrylamide gel electrophoresis (SDS-PAGE) and analyzed by protein gel blots using corresponding antibodies.

## Supporting Information

Figure S1 Loss of pTAC5 causes a heat-sensitive phenotype. (A) Gene structure of *At4g13670* showing the T-DNA insertion site of the SALK_049133. White boxes represent exons; thin lines indicate introns. Sequences of primers used for isolation of homozygous lines were indicated as follows: AtLB1∶5′-TGGTTCACGTAGTGGGCCATCG-3′; *At4g13670*-specific primers: 5′-TTAAGGAAGCTGGTAATGGGG-3′ and 5′-TTTTTCTTCTTACGAAAATAATATGCC-3′. (B) The expression of *pTAC5* in wild type and *ptac5* by semiquantitative reverse transcriptase (RT)-PCR analysis. The β-tublin was used as control. Primers used for RT-PCR analysis were as follows: β-tublin specific primers: 5′-GATTTCAAAGATTAGGGAAGAGTA-3′, 5′-GTTCTGAAGCAAATGTCATAGAG-3′; *pTAC5*-specific primers: 5′- CATATGATGTGCTTCTCCACTCAAAATC-3′ and 5′- GGATCCTTATAAGTTTTTTTTGCCGTC-3′. (C) Phenotype of *ptac5* mutants. Top panels show growth phenotype of *ptac5* mutants grown on MS medium for 7 days at 22°C compared with WT. Bottom panels show phenotypes of WT and *ptac5* seedlings after 7 days at 28°C.
(TIF)

## References

[1] von Arnim A, Deng XW (1996) Light control of seedling development. Annu Rev Plant Physiol Mol Biol 47: 215–243. 10.1146/annurev.arplant.47.1.215 15012288

[2] Shimada H, Mochizuki M, Ogura K, Froehlich JE, Osteryoung KW, et al (2007) *Arabidopsis* cotyledon-specific chloroplast biogenesis factor CYO1 is a protein disulfide isomerase. Plant Cell 19: 3157–3169. 17921316 10.1105/tpc.107.051714 PMC2174705

[3] Leister D (2003) Chloroplast research in the genomic age. Trends Genet 19: 47–56. 12493248 10.1016/s0168-9525(02)00003-3

[4] Albrecht V, Simkova K, Carrie C, Delannoy E, Giraud E, et al (2010) The cytoskeleton and the peroxisomal-targeted snowy cotyledon3 protein are required for chloroplast development in *Arabidopsis* . Plant Cell 22: 3423–3438. 20978221 10.1105/tpc.110.074781 PMC2990128

[5] Inaba T, Ito-Inaba Y (2010) Versatile roles of plastids in plant growth and development. Plant Cell Physiol 51: 1847–1853. 20889507 10.1093/pcp/pcq147

[6] Pogson BJ, Albrecht V (2011) Genetic dissection of chloroplast biogenesis and development: an overview. Plant Physiol 155: 1545–1551. 21330494 10.1104/pp.110.170365 PMC3091115

[7] Shiina T, Tsunoyama Y, Nakahira Y, Khan MS (2005) Plastid RNA polymerases, promoters, and transcription regulators in higher plants. Int Rev Cytol 244: 1–68. 16157177 10.1016/S0074-7696(05)44001-2

[8] Allison LA, Simon LD, Maliga P (1996) Deletion of *rpoB* reveals a second distinct transcription system in plastids of higher plants. EMBO J 15: 2802–2809. 8654377 PMC450217

[9] Hajdukiewicz PTJ, Allison LA, Maliga P (1997) The two RNA polymerases encoded by the nuclear and the plastid compartments transcribe distinct groups of genes in tobacco plastids. EMBO J 16: 4041–4048. 9233813 10.1093/emboj/16.13.4041 PMC1170027

[10] Igloi GL, Kössel H (1992) The transcriptional apparatus of chloroplasts. Crit Rev Plant Sci 10: 525–558.

[11] Suck R, Zeltz P, Falk J, Acker A, Kössel H, et al (1996) Transcriptionally active chromosomes (TACs) of barley chloroplasts contain the alpha-subunit of plastome-encoded RNA polymerase. Curr Genet 30: 515–521. 8939813 10.1007/s002940050164

[12] Hallick RB, Lipper C, Richards OC, Rutter WJ (1976) Isolation of a transcriptionally active chromosome from chloroplasts of Euglena gracilis. Biochemistry 15: 3039–3045. 821516 10.1021/bi00659a016

[13] Rushlow KE, Hallick RB (1982) The isolation and purification of a transcriptionally active chromosome from chloroplast of *Euglena gracilis*. In:Edelman M, Hallick RB, Chua NH, editors. Amsterdam: Elsevier Press. 543–550.

[14] Reiss T, Link G (1985) Characterization of transcriptionally active DNA-protein complexes from chloroplasts and etioplasts of mustard (*Sinapis alba L*.). Eur J Biochem 48: 207–212. 10.1111/j.1432-1033.1985.tb08826.x 2580705

[15] Pfalz J, Liere K, Kandlbinder A, Dietz KJ, Oelmuller R (2006) pTAC2, -6, and -12 are components of the transcriptionally active plastid chromosome that are required for plastid gene expression. Plant Cell 18: 176–197. 16326926 10.1105/tpc.105.036392 PMC1323492

[16] Myouga F, Hosoda C, Umezawa T, Iizumi H, Kuromori T, et al (2008) A heterocomplex of iron superoxide dismutases defends chloroplast nucleoids against oxidative stress and is essential for chloroplast development in *Arabidopsis* . Plant Cell 20: 3148–3162. 18996978 10.1105/tpc.108.061341 PMC2613658

[17] Arsova B, Hoja U, Wimmelbacher M, Greiner E, Ustun S, et al (2010) Plastidial thioredoxin z interacts with two fructokinase-like proteins in a thiol-dependent manner: evidence for an essential role in chloroplast development in *Arabidopsis* and *Nicotiana benthamiana* . Plant Cell 22: 1498–1515. 20511297 10.1105/tpc.109.071001 PMC2899873

[18] Steiner S, Schroter Y, Pfalz J, Pfannschmidt T (2011) Identification of essential subunits in the plastid-encoded RNA polymerase complex reveals building blocks for proper plastid development. Plant Physiol 157: 1043–1055. 21949211 10.1104/pp.111.184515 PMC3252157

[19] Gilkerson J, Perez-Ruiz JM, Chory J, Callis J (2012) The plastid-localized pfkB-type carbohydrate kinases FRUCTOKINASE-LIKE 1 and 2 are essential for growth and development of *Arabidopsis thaliana*, BMC Plant Biol. 12: 102. 10.1186/1471-2229-12-102 PMC3409070 22770232

[20] Yagi Y, Ishizaki Y, Nakahira Y, Tozawa Y, Shiina T (2012) Eukaryotic-type plastid nucleoid protein pTAC3 is essential for transcription by the bacterial-type plastid RNA polymerase. Proc Natl Acad Sci U S A 109: 7541–7546. 22529394 10.1073/pnas.1119403109 PMC3358912

[21] Yu QB, Lu Y, Ma Q, Zhao TT, Huang C, et al. (2012) TAC7, an essential component of the plastid transcriptionally active chromosome complex, interacts with FLN1, TAC10, TAC12 and TAC14 to regulate chloroplast gene expression in *Arabidopsis thaliana*. Physiol Plant doi:10.1111/j.1399-3054.2012.01718.x. 10.1111/j.1399-3054.2012.01718.x 23082802

[22] Gao ZP, Yu QB, Zhao TT, Ma Q, Chen GX, et al (2011) A functional component of the transcriptionally active chromosome complex, Arabidopsis pTAC14, interacts with pTAC12/HEMERA and regulates plastid gene expression. Plant Physiol 157: 1733–1745. 22010110 10.1104/pp.111.184762 PMC3327189

[23] Yamamoto YY, Puente P, Deng XW (2000) An *Arabidopsis* cotyledon-specific albino locus: a possible role in 16S rRNA maturation. Plant Cell Physiol 41: 68–76. 10750710 10.1093/pcp/41.1.68

[24] Chi W, Ma JF, Zhang DY, Guo JK, Chen F, et al (2008) The pentratricopeptide repeat protein DELAYED GREENING1 is involved in the regulation of early chloroplast development and chloroplast gene expression in *Arabidopsis* . Plant Physiol 147: 573–584. 18400937 10.1104/pp.108.116194 PMC2409026

[25] Shipman-Roston RL, Ruppel NJ, Damoc C, Phinney BS, Inoue K (2010) The significance of protein maturation by plastidic type I signal peptidase 1 for thylakoid development in *Arabidopsis* chloroplasts. Plant Physiol 152: 1297–1308. 20097790 10.1104/pp.109.151977 PMC2832241

[26] Zhou WB, Cheng YX, Yap A, Laure A, Boutin C, et al (2009) The *Arabidopsis* gene *YS1* encoding a DYW protein is required for editing of *rpoB* transcripts and the rapid development of chloroplasts during early growth. Plant J 58: 82–96. 19054358 10.1111/j.1365-313X.2008.03766.x

[27] Cao ZL, Yu QB, Sun Y, Lu Y, Cui YL, et al (2011) A point mutation in the pentatricopeptide repeat motif of the AtECB2 protein causes delayed chloroplast development. J Integr Plant Biol 53: 258–269. 21294841 10.1111/j.1744-7909.2011.01030.x

[28] Fromme P, Melkozernov A, Jordan P, Krauss N (2003) Structure and function of photosystem I: Interaction with its soluble electron carriers and external antenna systems. FEBS Lett 555: 40–44. 14630316 10.1016/s0014-5793(03)01124-4

[29] Barnes SA, Nishizawa NK, Quaggio RB, Whitelam GC, Chua NH (1996) Far-red light blocks greening of arabidopsis seedlings via a phytochrome A-mediated change in plastid development. Plant Cell 8: 601–615. 8624438 10.1105/tpc.8.4.601 PMC161123

[30] Hudson D, Guevara D, Yaish MW, Hannam C, Long N, et al (2011) *GNC* and *CGA1* modulate chlorophyll biosynthesis and glutamate synthase (*GLU1/Fd-GOGAT*) expression in *Arabidopsis* . PLoS ONE 6: e26765. 22102866 10.1371/journal.pone.0026765 PMC3213100

[31] Nott A, Jung HS, Koussevitzky S, Chory J (2006) Plastid-to-nucleus retrograde signaling. Annu Rev Plant Biol 57: 739–759. 16669780 10.1146/annurev.arplant.57.032905.105310

[32] Tang WJ, Wang WQ, Chen DQ, Ji Q, Jing YJ, et al (2012) Transposase-derived proteins FHY3/FAR1 interact with PHYTOCHROME-INTERACTING FACTOR1 to regulate chlorophyll biosynthesis by modulating *HEMB1* during deetiolation in *Arabidopsis* . Plant Cell 24: 1984–2000. 22634759 10.1105/tpc.112.097022 PMC3442582

[33] Zhong SW, Zhao MT, Shi TY, Shi H, An FY, et al (2009) EIN3/EIL1 cooperate with PIF1 to prevent photo-oxidation and to promote greening of *Arabidopsis* seedlings. Proc Natl Acad Sci U S A 106: 21431–21436. 19948955 10.1073/pnas.0907670106 PMC2795496

[34] Waters MT, Wang P, Korkaric M, Capper RG, Saunders NJ, et al (2009) GLK transcription factors coordinate expression of the photosynthetic apparatus in *Arabidopsis* . Plant Cell 21: 1109–1128. 19376934 10.1105/tpc.108.065250 PMC2685620

[35] Kobayashi K, Baba S, Obayashi T, Sato M, Toyooka K, et al (2012) Regulation of root greening by light and auxin/cytokinin signaling in *Arabidopsis* . Plant Cell 24: 1081–1095. 22415275 10.1105/tpc.111.092254 PMC3336121

[36] Chi W, Mao J, Li QN, Ji DL, Zou ML, et al (2010) Interaction of the pentatricopeptide-repeat protein DELAYED GREENING 1 with sigma factor SIG6 in the regulation of chloroplast gene expression in *Arabidopsis* cotyledons. Plant J 64: 14–25. 20626654 10.1111/j.1365-313X.2010.04304.x

[37] Ogawa T, Nishimura K, Aoki T, Takase H, Tomizawa K, et al (2009) A phosphofructokinase B-type carbohydrate kinase family protein, NARA5, for massive expressions of plastid-encoded photosynthetic genes in *Arabidopsis* . Plant Physiol 151: 114–128. 19587101 10.1104/pp.109.139683 PMC2736000

[38] Zhelyazkova P, Sharma CM, Förstner KU, Liere K, Vogel J, et al (2012) The primary transcriptome of barley chloroplasts: numerous noncoding RNAs and the dominating role of the plastid-encoded RNA polymerase. Plant Cell 24: 123–136. 22267485 10.1105/tpc.111.089441 PMC3289561

[39] Link G (2003) Redox regulation of chloroplast transcription. Antioxid Redox Signal 5: 79–87. 12626119 10.1089/152308603321223568

[40] Dietz KJ, Pfannschmidt T (2011) Novel regulators in photosynthetic redox control of plant metabolism and gene expression. Plant Physiol 155: 1477–1485. 21205617 10.1104/pp.110.170043 PMC3091116

[41] Yu QB, Jiang Y, Chong K, Yang ZN (2009) AtECB2, a pentatricopeptide repeat protein, is required for chloroplast transcript *accD* RNA editing and early chloroplast biogenesis in *Arabidopsis thaliana* . Plant J 59: 1011–1023. 19500301 10.1111/j.1365-313X.2009.03930.x

[42] Motohashi R, Nagata N, Ito T, Takahashi S, Hobo T, et al (2001) An essential role of a TatC homologue of a Delta pH dependent protein transporter in thylakoid membrane formation during chloroplast development in *Arabidopsis thaliana* . Proc Natl Acad Sci U S A 98: 10499–10504. 11526245 10.1073/pnas.181304598 PMC56989

