# Supplementary figures and images for: The Reduced Plastid-Encoded Polymerase-Dependent Plastid Gene Expression Leads to the Delayed Greening of the Arabidopsis fln2 Mutant
Source: PLoS One. 2013 Sep 3;8(9):e73092. doi: 10.1371/journal.pone.0073092 (PMC3760890; doi:10.1371/journal.pone.0073092)

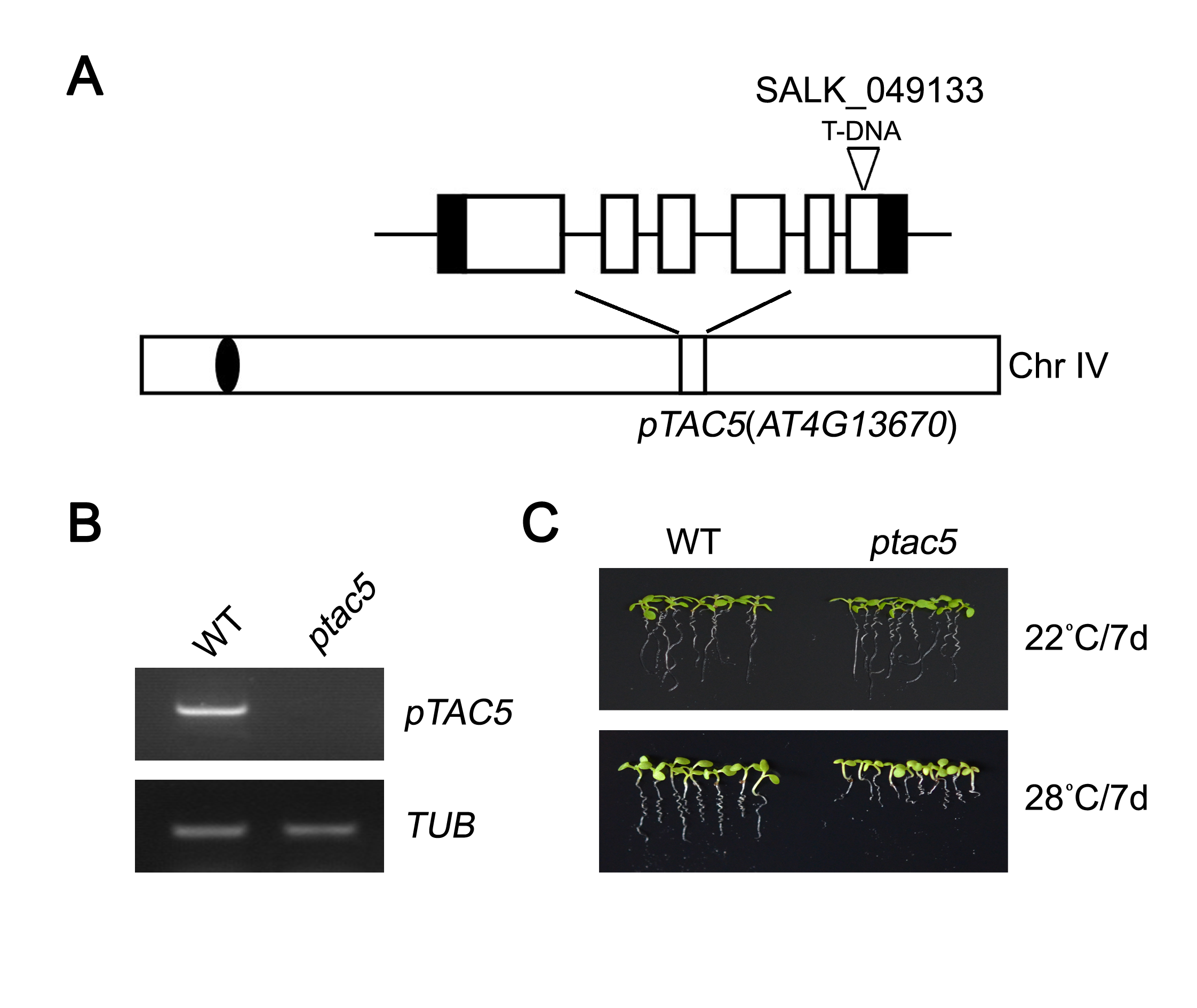

Supplement: Figure S1 — Loss of pTAC5 causes a heat-sensitive phenotype. (A) Gene structure of At4g13670 showing the T-DNA insertion site of the SALK_049133. White boxes represent exons; thin lines indicate introns. Sequences of primers used for isolation of homozygous lines were indicated as follows: AtLB1∶5′-TGGTTCACGTAGTGGGCCATCG-3′; At4g13670-specific primers: 5′-TTAAGGAAGCTGGTAATGGGG-3′ and 5′-TTTTTCTTCTTACGAAAATAATATGCC-3′. (B) The expression of pTAC5 in wild type and ptac5 by semiquantitative reverse transcriptase (RT)-PCR analysis. The β-tublin was used as control. Primers used for RT-PCR analysis were as follows: β-tublin specific primers: 5′-GATTTCAAAGATTAGGGAAGAGTA-3′, 5′-GTTCTGAAGCAAATGTCATAGAG-3′; pTAC5-specific primers: 5′- CATATGATGTGCTTCTCCACTCAAAATC-3′ and 5′- GGATCCTTATAAGTTTTTTTTGCCGTC-3′. (C) Phenotype of ptac5 mutants. Top panels show growth phenotype of ptac5 mutants grown on MS medium for 7 days at 22°C compared with WT. Bottom panels show phenotypes of WT and ptac5 seedlings after 7 days at 28°C. (TIF) [file pone.0073092.s001.tif]
